# Cumulative pre-chemoimmunotherapy dexamethasone dose is associated with adverse outcomes in primary central nervous system lymphoma

**DOI:** 10.3389/fonc.2026.1905984

**Published:** 2026-09-17

**Authors:** Artem Rafaelian, Sae-Yeon Won, Gleb Dashevskii, Felix M. Gerach, Laurèl Rauschenbach, Tatiana Chacón-Quesada, Silvia Hernández Durán, Paulina Guenzerodt, Peter Baumgarten, Dorothee Mielke, Veit Rohde, Ulrich Sure, Henry Schroeder, Claudia Maletzki, Ulrich Langenkamp, Sebastian Böttcher, Christian Junghanss, Bastian von Tresckow, Thomas M. Freiman, Florian Gessler, Daniel Dubinski

**Affiliations:** 1 Department of Neurosurgery, Rostock University Medical Center, Rostock, Germany; 2 Department of Neurosurgery, University Medicine Greifswald, Greifswald, Germany; 3 Department of Neurosurgery and Spine Surgery, University Hospital Essen, Essen, Germany; 4 Center for Translational Neuroscience and Behavioral Science, University of Duisburg-Essen, Essen, Germany; 5 DKFZ Division of Translational Neurooncology at the West German Cancer Center (WTZ), German Cancer Consortium (DKTK), Partner Site Essen/Düsseldorf, A Partnership between DKFZ and University Hospital Essen, University Duisburg-Essen, Essen, Germany; 6 Department of Neurosurgery, University Medical Center Göttingen, Göttingen, Germany; 7 Department of Neurosurgery, University Hospital Augsburg, Augsburg, Germany; 8 Department of Internal Medicine - Clinic and Polyclinic for Hematology, Hemostaseology, Oncology, Stem Cell Therapy and Palliative Medicine, Rostock University Medical Center, Rostock, Germany; 9 Department of Hematology and Stem Cell Transplantation, West German Cancer Center and German Cancer Consortium (DKTK partner site Essen), University Hospital Essen, University of Duisburg-Essen, Essen, Germany

**Keywords:** dexamethasone, lymphoma, outcome, overall survival (OS), PCNSL

## Abstract

**Introduction:**

Dexamethasone is administered postoperatively and concomitantly with induction regimens and consolidation therapy in patients with primary central nervous system lymphoma (PCNSL). This study aimed to evaluate the impact of cumulative dexamethasone administration prior to systemic chemoimmunotherapy on overall survival (OS) and progression-free survival (PFS) in patients with PCNSL.

**Methods:**

We conducted a multicenter retrospective study of 105 patients with newly diagnosed, histologically confirmed PCNSL who underwent chemoimmunotherapy between 2014 and 2024. Patients were dichotomized according to cumulative dexamethasone exposure prior to chemoimmunotherapy using an exploratory threshold of 154 mg determined by the maximally selected log-rank statistic.

**Results:**

High cumulative dexamethasone exposure group (HDE, >154 mg) was associated with significantly worse OS (*p* <.001) and PFS (*p* <.001) compared with the low cumulative dexamethasone exposure group. In univariate analysis, HDE was significantly associated with poor outcome and increased mortality (*p* = .0001), whereas other clinical variables such as Karnofsky Performance Status (*p* = .800), ASA score (*p* = .052), and tumor volume (*p* = .778) did not show significant associations. In multivariate Cox regression, cumulative dexamethasone exposure remained independently associated with poorer survival (*p* = .002), while autologous stem cell transplantation was associated with improved survival (*p* = .002).

**Conclusion:**

HDE prior to chemoimmunotherapy was independently associated with worse survival outcomes in patients with PCNSL. A cumulative dexamethasone dose of 154 mg was identified as an exploratory threshold associated with survival differences and requires prospective validation before clinical implementation.

## Introduction

Primary central nervous system lymphoma (PCNSL) is a rare extranodal variant of non-Hodgkin lymphoma arising within the central nervous system. With an annual incidence of approximately 0.44 per 100,000, PCNSL represents the third most common primary malignant brain tumor after glioblastoma and astrocytoma of varying grades (1). According to the 5th edition of the World Health Organization Classification of Hematolymphoid Tumors, PCNSL is classified as a primary large B-cell lymphoma of immunoprivileged sites (2–5). Over the past decades, outcomes in PCNSL have markedly improved with the introduction of high-dose methotrexate (HD-MTX)–based chemoimmunotherapy, consolidation strategies including high-dose chemotherapy followed by autologous stem cell transplantation, and the addition of monoclonal antibodies such as rituximab (6). As a result, median overall survival has increased from approximately 2.5 months to more than 2 years (7).

Despite these advances, the role of neurosurgery in PCNSL remains largely diagnostic. Although surgical resection has been increasingly reported in selected cases, meta-analyses have not demonstrated a clear survival benefit, and stereotactic biopsy remains the standard approach for histopathological confirmation (8–10). Consequently, most therapeutic optimization efforts have focused on systemic treatment strategies (11). In routine neurosurgical practice, however, perioperative administration of dexamethasone remains common. Corticosteroids are frequently prescribed to reduce cerebral edema, improve neurological symptoms, and stabilize patients prior to initiation of systemic therapy. In hematologic oncology, corticosteroid prephase therapy has been shown to improve treatment tolerability in aggressive peripheral B-cell lymphomas, particularly in elderly patients (12, 13). In PCNSL, by contrast, multiple studies have demonstrated that preoperative corticosteroid administration should generally be avoided because of an increased risk of false-negative biopsy results, and occasionally necessitate repeat biopsy to establish a definitive diagnosis (8, 14). PCNSL cells are highly susceptible to corticosteroid-induced apoptosis, which may result in marked depletion of neoplastic B cells and biopsy specimens dominated by reactive and resorptive changes (15, 16). Beyond diagnostic considerations, emerging evidence suggests that excessive corticosteroid exposure may adversely affect outcomes in several CNS malignancies (17–20).

The impact of dexamethasone administration prior to chemotherapy has not been specifically investigated in PCNSL, and its prognostic significance remains unclear. Taken together, these observations highlight an unmet need to clarify the prognostic significance of dexamethasone administration prior to chemotherapy. Therefore, this study aimed to determine whether cumulative dexamethasone exposure prior to chemotherapy independently associated with verall and progression-free survival in patients with PCNSL.

## Methods

### Patient selection

Between 2014 and 2024, a total of 105 patients with newly diagnosed, histologically confirmed PCNSL who underwent neurosurgical intervention (stereotactic biopsy or resection) at 5 participating institutions were included. All patients with newly diagnosed, histologically verified PCNSL presenting between 2014 and 2024 were retrospectively entered into a curated institutional database. Given the non-interventional, multicenter, retrospective design, the requirement for individual informed consent was waived. This study complied with the Declaration of Helsinki (21).

Exclusion criteria were absence of preoperative MRI or CT data for volumetric analysis, lack of histopathological confirmation, prior hematologic malignancy, chronic corticosteroid use before hospitalization, or incomplete documentation of dexamethasone administration or chemoimmunotherapy regimens.

### Data collection

Clinical, demographic, radiological, and treatment-related data were extracted from electronic medical records and follow-up documentation. Collected variables included age, sex, comorbidities, anticoagulation status, Karnofsky Performance Status (KPS), ASA classification, body surface area (BSA), tumor size and location, extent of surgical intervention, perioperative complications, and details of systemic therapy.

The Memorial Sloan Kettering Cancer Center (MSKCC) prognostic score was calculated based on age and KPS at diagnosis (Class 1: age<50 years; Class 2: age ≥50 years and KPS ≥70; Class 3: age ≥50 years and KPS<70) (22). Cumulative pre-chemoimmunotherapy dexamethasone exposure was calculated as the total documented dose administered from first corticosteroid use until initiation of systemic therapy, including preoperative, postoperative, and, where applicable, inter-biopsy treatment. In patients requiring repeat biopsy, dexamethasone administered between the initial and definitive diagnostic procedures was included in the cumulative pre-chemoimmunotherapy exposure. Dose adjustments were incorporated, and treatment duration was recorded separately. Patients who did not receive dexamethasone before initiation of systemic therapy were assigned a cumulative exposure of 0 mg, representing a valid value of the cumulative exposure variable, and were retained in the primary analyses. Preoperative tumor volume was assessed on contrast-enhanced T1-weighted MRI using the ellipsoid $\text{formula}=\frac{\text{A}\times \text{B}\times \text{C}}{2}$ where A, B, and C represent the largest orthogonal diameters measured manually (23). T2-weighted images were reviewed to evaluate edema and midline shift, and postoperative CT scans were assessed for hemorrhagic complications. Overall survival (OS) was defined as the interval from histological diagnosis to death from any cause. Progression-free survival (PFS) was defined as the interval from diagnosis to radiological or clinical progression according to standard response criteria. Patients without an event were censored at last follow-up. Median follow-up was 23 months (IQR 10–50).

PCNSL diagnosis was established by institutional neuropathological examination integrating histomorphological and immunophenotypic findings. Available histomorphological, immunophenotypic, and immunohistochemical findings, including Ki-67 proliferation indices, are presented in **Supplementary Table S1** together with the corresponding pathological diagnoses.

### Statistical analyses

Continuous variables are presented as medians with interquartile ranges (IQR), and categorical variables as counts and percentages. To explore the prognostic relevance of cumulative pre-chemoimmunotherapy dexamethasone exposure, an exploratory cut-off was identified using the maximally selected log-rank statistic implemented in Python (v3.11, statsmodels package) (24) Statistical significance was verified using a 2000-fold permutation test. Survival distributions were estimated using the Kaplan–Meier method and compared with the log-rank test. Cox proportional hazards regression was applied to identify independent predictors of outcome. Variables with p<.10 in univariable analysis and clinically relevant potential confounders were included in the multivariable Cox regression model. Tumor volume, dexamethasone treatment duration, and the interval between surgery and chemoimmunotherapy were included irrespective of their univariable p values because of their potential association with dexamethasone exposure and outcome. Results are reported as hazard ratios (HRs) with 95% confidence intervals (CIs). For secondary analyses, patients were categorized into a remission group (RG; complete remission according to RANO criteria) and a deceased group (DG) (25) Group comparisons were performed using the Mann–Whitney U test for continuous variables and χ^2^ or Fisher’s exact test for categorical variables. All tests were two-sided, and p< 0.05 was considered statistically significant. Statistical analyses were performed using SPSS v22 (IBM, Armonk, NY, USA) and GraphPad Prism v10.

### Systemic therapy classification

Systemic therapy was administered according to institutional protocols and physician discretion. Regimens were categorized into three groups based on composition and intensity: R-MTX (base) - standard rituximab and methotrexate-based protocols, including combinations with procarbazine, typically used in older or less fit patients. Alternative regimens - intensified or modified protocols including additional cytotoxic agents (e.g., ifosfamide, cytarabine, thiotepa), primarily used in patients eligible for consolidative high-dose chemotherapy and autologous stem cell transplantation, consistent with current EHA–ESMO guidelines (26–28). Other regimens — heterogeneous or non-standard combinations. Rituximab-containing therapy was administered in 102 of 105 patients (97.1%). The three patients who did not receive rituximab were classified within the “Other” treatment category. Complications related to systemic chemoimmunotherapy included MTX-associated acute renal failure, sepsis, fatigue with subsequent refusal of therapy, and pulmonary embolism. In several cases, these adverse events required temporary interruption or postponement of planned chemoimmunotherapy cycles.

## Results

### Baseline characteristics

The median age of the cohort was 68 years (IQR 61–74), and 55 patients (52.4%) were male. Median body surface area was 1.9 m^2^ (IQR 1.8–2.1). Stereotactic biopsy was performed in 92 patients (87.6%). Median preoperative ASA score was 2 (IQR 2–3), and median Karnofsky Performance Status (KPS) was 70 (IQR 60–90). A single lesion was identified in 63 patients (60%), while 42 patients (40%) presented with multiple lesions. Median tumor volume was 15.4 cm^3^ (IQR 8.0–27.6). Cumulative pre-chemoimmunotherapy dexamethasone exposure was >0 mg in 100 patients (95.2%), whereas five patients (4.8%) received no dexamethasone before initiation of systemic therapy and therefore had a cumulative exposure of 0 mg. All cases were histologically confirmed as diffuse large B-cell lymphoma of the CNS according to institutional neuropathological assessment, with a median Ki-67 index of 80% (IQR 70–90) (3). At last follow-up, 38 patients (36.2%) had died. One- and two-year overall survival rates were 83.8% and 76.2%, respectively (**Table 1**).

**Table 1:** Baseline clinical characteristics of the study cohort at the time of diagnosis, including subgroup division into low cumulative and high cumulative dexamethasone exposure group.

Patient characteristic	Cohort	Low cumulative dexamethasone exposure group (≤154 mg)	High cumulative dexamethasone exposure group (>154 mg)	Univariate	
	Count 105	54	51	OR 95% CI	p - value
Sex, male, n (%)	55 (52.4)	29 (53.7)	26 (50.9)	1.11 (0.51 - 2.40)	.466
Age, median (IQR)	68 (61-74)	69 (62 - 74)	67 (61 -75)	–	.869
BSA, median (IQR)	1.9 (1.8 - 2.1)	2.0 (1.8 - 2.1)	1.9 (1.8 - 2.1)	–	.952
Operation type					
Stereotactic biopsy	92 (87.6)	48 (88.9)	44 (86.3)	1.27 (0.39 - 4.07)	.455
Open biopsy	4 (3.8)	1 (1.9)	3 (5.9)	0.30 (0.03 - 3.01)	.287
Resection	9 (8.6)	5 (9.2)	4 (7.8)	1.19 (0.30 -4.74)	.536
Re-biopsy	8 (7.6)	3 (5.5)	5 (9.8)	0.54 (0.12 - 2.39)	.326
Preoperative ASA, median (IQR)	2 (2-3)	2 (2-3)	3 (2-3)	–	**.019**
1-2, n (%)	51 (48.6)	32 (59.3)	19 (37.3)	2.44 (1.11 - 5.37)	**.019**
3-4, n (%)	54 (51.4)	22 (40.7)	32 (62.7)	0.41 (0.18 - 0.89)	**.019**
Preoperative KPS, median (IQR)	70 (60 - 90)	70 (60-90)	70 (70 - 90)	–	.596
100-80, n (%)	47 (44.8)	22 (40.7)	25 (49)	0.71 (0.33 -1.54)	.255
70-60, n (%)	46 (43.8)	28 (51.9)	18 (35.3)	1.97 (0.90 -4.32)	.064
<60, n (%)	12 (11.4)	4 (7.4)	8 (15.7)	0.43 (0.12 - 1.52)	.152
MSKCC prognostic score median, (IQR)	2 (2 - 3)	2 (2 - 3)	2 (2 - 2)	–	.784
class 1, n (%)	7 (6.7)	4 (7.4)	3 (5.9)	1.28 (0.27 - 6.02)	.532
class 2, n (%)	71 (67.6)	35 (64.8)	36 (70.6)	0.76 (0.33 - 1.74)	.336
class 3, n (%)	27 (25.7)	15 (27.8)	12 (23.5)	1.25 (0.52 - 3.01)	.392
Ki67, median (IQR)	80 (70 - 90)	80 (70 - 80)	80 (70 - 90)	–	.057
90-80%, n (%)	62 (59)	28 (51.9)	34 (66.7)	0.53 (0.24 - 1.18)	.089
60-70%, n (%)	38 (36.2)	23 (42.6)	15 (29.4)	1.78 (0.79 - 3.99)	.114
<60%, n (%)	5 (4.8)	3 (5.5)	2 (3.9)	0.46 (0.04 - 5.26)	.478
History of other malignancies, n (%)	19 (18.1)	10 (18.5)	9 (17.6)	1.06 (0.39 - 2.86)	.555
Diabetes mellitus, n (%)	23 (21.9)	11 (20.4)	12 (23.5)	0.83 (0.32 - 2.09)	.438
Hypertension, n (%)	65 (61.9)	36 (66.6)	29 (56.9)	1.51 (0.68 - 3.34)	.202
Anticoagulation, n (%)	11 (10.5)	6 (11.1)	5 (9.8)	1.15 (0.32 - 4.02)	.541
Antiplatelet use, n (%)	19 (18.1)	7 (12.7)	12 (23.5)	0.48 (0.17 - 1.34)	.124
Tumor volume (mL), median (IQR)	15.4 (8 -27.6)	12.8 (4.3 - 19.0)	20 (11 - 37)	–	**.005**
Peritumoral edema (mL), median (IQR)	64.9 (25.2 - 120.1)	68.0 (28.8 - 120.5)	60.1 (21.3 - 113.3)	–	.824
Total number of lesions, median (IQR)	1 (1 - 2)	1 (1 - 2.8)	1 (1 -2)	–	.134
Midline shift (mm), median (IQR),	2 (0 - 4.3)	1.5 (0 - 4.0)	2 (0 - 5.0)	–	.243
Location					
supratentorial	95 (90.5)	48 (88.8)	47 (92.2)	0.68 (0.18 - 2.18)	.407
infratentorial	1 (0.9)	0 (0)	1 (1.9)	–	.485
both	9 (8.6)	6 (11.2)	3 (5.9)	2 (0.47 - 8.46)	.273
Intracerebral hemorrhage on postoperative CT, n (%)	58 (55.2)	28 (51.9)	30 (58.8)	0.75 (0.68 - 1.34)	.301
Revision surgery, n (%)	2 (1.9)	1 (1.9)	1 (1.9)	0.94 (0.05 - 15.49)	.737
Non-space-occupying, n(%)	56 (53.3)	27 (50)	29 (56.9)	0.75 (0.35 - 1.63)	.305
Abscess or impaired wound healing, n (%)	3 (2.9)	1 (1.9)	2 (3.9)	0.17 (0.01 - 1.54)	.09
Preoperative Dex, n (%)	21 (20)	11 (20.4)	10 (19.6)	1.04 (0.41 - 2.73)	.558
Postoperative Dex before systemic therapy, n (%)	98 (93.3)	49 (90.7)	49 (96.1)	0.4 (0.07 - 2.16)	.242
Duration of Dexamethasone administration (days) median (IQR)	11 (7-15)	8 (3 -12)	14 (12 - 19)	–	**<.0001**
Systemic therapy					
R-MTX-base	33 (31.4)	17 (31.5)	16 (31.4)	1.01 (0.44 - 2.29)	.578
Alternative regimens (intensive)	68 (64.8)	34 (63)	34 (66.7)	0.85 (0.38 - 1.89)	.424
Other	4 (3.8)	3 (5.5)	1 (1.9)	2.94 (0.29 - 29.23)	.330
Time between operation and chemo-immunotherapy (days) median (IQR)	13 (9-18)	11 (8 - 16)	14 (12 - 20)	–	**<.0001**
Complications, n (%)	40 (38.1)	19 (35.2)	21 (41.2)	0.77 (0.35 - 1.70)	.333
Stem cell transplantation, n (%)	26 (24.7)	16 (29.6)	10 (19.6)	1.72 (0.69 - 4.26)	.167
1-year OS, n (%)	88 (83.8)	51 (94.4)	37 (72.5)	–	**.002**
2-year OS, n (%)	80 (76.2)	51 (94.4)	30 (58.8)	–	**<.0001**
Deceased cases, n (%)	38 (36.2)	8 (14.8)	30 (58.8)	0.12 (0.04 - 0.31)	**<.0001**

Systemic therapy was classified into three categories: R-MTX (base) - rituximab plus methotrexate ± procarbazine; Alternative regimens - intensive or modified protocols including ifosfamide, cytarabine, or thiotepa; Other regimens - heterogeneous or non-standard combinations ratio, ASA, American Society of Anesthesiologists; MSKCC, Memorial Sloan-Kettering Cancer Center; BSA, body surface area; DEX, dexamethasone; IQR, interquartile range; KPS, Karnofsky Performance Status; MTX, methotrexate; OR, odds ratio; OS, overall survival.

Highlighted in bold: statistically significant, p < 0.05.

### Cumulative low cumulative dexamethasone exposure group versus high cumulative

Using maximally selected log-rank statistics, an exploratory threshold of 154 mg cumulative dexamethasone prior to chemoimmunotherapy was identified as optimally discriminating overall survival (χ^2^ = 21.16; permutation p = .002). Patients were classified into a LDE, (≤154 mg; n = 54) and a HDE (>154 mg; n = 51). Baseline characteristics were largely comparable between groups. Median age (69 vs. 67 years; p = .869), BSA (p = .952), type of surgical procedure (p = .455), comorbidities, KPS (p = .596), and systemic therapy regimens did not differ significantly. ASA 1–2 status was more frequent in the LDE group (59.3% vs. 37.3%; p = .019), and tumor volume was significantly larger in the HDE group (median 20.0 vs. 12.8 cm^3^; p = .005).

In contrast, there was no significant difference in preoperative KPS, which was 70 (IQR 60-90) in the LDE group and 70 (IQR 70-90) in the HDE group (*p* = .596). A significant difference was observed in tumor volume, with a median of 12.8 cm^3^ (IQR 4.3-19.0) in the LDE group versus 20.0 cm^3^ (IQR 11.0-37.0) in the HDE group (*p* = .005).

A significant difference in survival rates was observed between the two groups. The 1-year OS rate was 94.4% in the LDE compared with 72.5% in the HDE group (OR = 0.15, 95% CI 0.04-0.58, *p* = .002). The 2-year OS rate was 94.4% versus 58.8%, respectively (OR = 0.08, 95% CI 0.02-0.30, *p* <.001) (**Table 1**).

### Impact of pre-chemoimmunotherapy dexamethasone exposure on survival

Kaplan–Meier analysis demonstrated significantly worse overall survival and progression free survival in the HDE group compared with the LDE group (p<.001). (**Figure 1**). Cox proportional hazards analysis demonstrated a higher hazard of death in the HDE group (HR 5.80, 95% CI 2.41–13.96, p<.001) and a higher hazard of progression or death (HR 4.05, 95% CI 1.99–8.27, p<.001).

**Figure 1:**
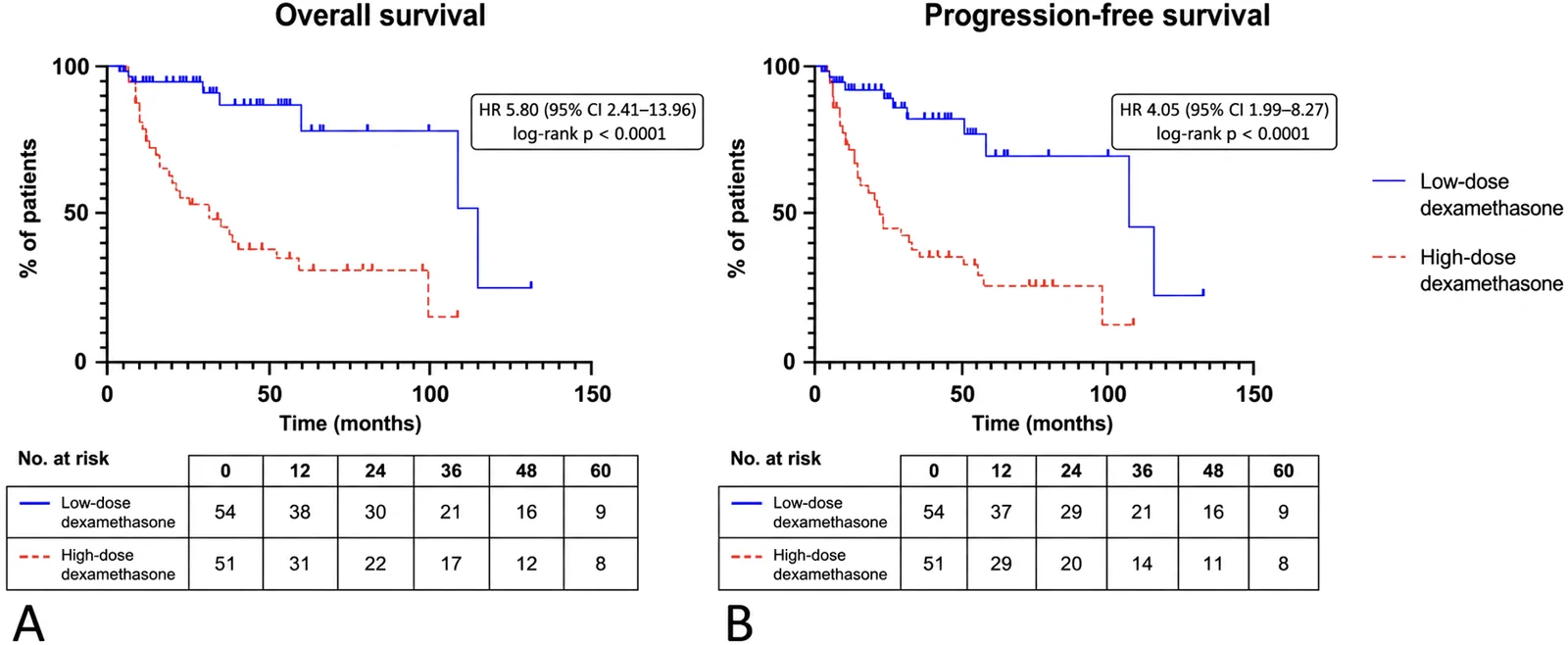
Kaplan-Meier analysis of overall survival (OS) and progression-free survival (PFS) according to dexamethasone exposure. **(A)** OS curves demonstrating significantly shorter survival in the HDE group (*p* <.001). **(B)** PFS curves showing a similar trend (*p* <.001).

### Exploratory analysis of patients without dexamethasone exposure

Five patients had no dexamethasone exposure before initiation of chemoimmunotherapy (0 mg). In an exploratory sensitivity analysis, these patients were compared with 49 steroid-exposed patients within the low cumulative exposure range (1–154 mg). No significant differences were observed in 1-year OS und 2-year OS (p = .257). Baseline and treatment characteristics were largely comparable, although tumor volume was lower in the unexposed group (median 3.9 vs. 14.6 mL; p = .032). Detailed comparisons are provided in **Supplementary Tables S2**.

### Risk factors for poor outcome

Patients were dichotomized into a remission group (RG) (n = 47; 44.8%) and a deceased group (DG) (n = 38; 36.2%) to evaluate clinical factors associated with treatment outcomes, 20 living patients without documented complete remission at the time of analysis were not included in this subgroup analysis. Age (*p* = .545), surgical procedure (*p* = .152), KPS (*p* = .164), MSKCC score (*p* = .225), tumor volume (*p* = .721), type of systemic therapy (*p* = .419) and time to initiation of chemoimmunotherapy (*p* = .363) did not differ significantly between groups.

A lower preoperative ASA score was associated with survival: ASA 1–2 was present in 25 patients (53.2%) in the RG versus 13 patients (34.2%) in the DG (OR 0.39, 95% CI 0.17-0.91, *p* = .069). In multivariate Cox regression, several independent predictors of survival were identified. Autologous stem cell transplantation was associated with improved survival (performed in 19 patients (40.4%) in the RG vs. 3 (7.9%) in the DG; adjusted HR 0.06, 95% CI 0.01-0.37, *p* = .002). Conversely, a higher cumulative perioperative dexamethasone dose prior to systemic therapy was independently associated with worse survival (median 120 mg (IQR 66-168) in survivors vs. 184 mg (IQR 157-233) in non-survivors; HR 1.2, 95% CI 1.05-1.2, *p* = .002). No other variables, including tumor volume, type of chemotherapy, perioperative complications, BSA, KPS, ASA or time interval of dexamethasone therapy, retained statistical significance in the multivariate model (**Table 2**).

**Table 2:** Comparison of clinical and treatment characteristics between survivors and non-survivors.

Patient characteristic	Remission group	Deceased group	Univariate		Multivariate	
	47	38	OR 95% CI	p - value	HR 95% CI	p - value
Sex, male, n (%)	27 (57.4)	19 (50)	1.35 (0.57 - 3.18)	.639	0.96 (0.32 - 2.89)	.942
Age, median (IQR)	67 (58.5 - 73)	68 (61 - 72)		.545	0.96 (0.90 - 1.03)	.278
BSA, median (IQR)	2.0 (1.8 - 2.1)	2.0 (1.8 - 2.1)		.488		
Operation type						
Stereotactic biopsy	43 (91.5)	31 (81.5)	2.42 (0.65 - 9.02)	.152		
Open biopsy	1 (2.1)	2 (5.3)	0.39 (0.04 - 4.48)	.419		
Resection	3 (6.4)	5 (13.2)	0.45 (0.11 - 2.02)	.457		
Re-biopsy	3 (6.4)	2 (5.3)	1.22 (0.19 - 7.74)	.601		
Preoperative ASA, median (IQR)	2 (2 - 3)	3 (2 - 3)		.069	2.32 (0.73 - 7.41)	.153
1-2, n (%)	25 (53.2)	13 (34.2)	2.18 (0.91 - 5.27)	.062		
3-4, n (%)	22 (46.8)	25 (65.8)	0.45 (0.19 - 1.11)	.062		
Preoperative KPS, median (IQR)	80 (70 - 90)	70 (70 - 90)		.164	0.98 (0.94 - 1.03)	.457
100-80, n (%)	24 (51.1)	15 (39.5)	1.6 (0.67 - 3.8)	.396		
70-60, n (%)	20 (42.5)	16 (42.1)	1.01 (0.42 - 2.42)	.571		
<60, n (%)	3 (6.4)	7 (18.4)	0.31 (0.07 - 1.26)	.084		
MSKCC prognostic score median, (IQR)	2 (2-2)	2 (2-3)		.225		
class 1, n (%)	5 (10.6)	2 (5.3)	2.02 (0.41 - 9.84)	.313		
class 2, n (%)	32 (68.1)	24 (63.2)	1.24 (0.51 - 3.07)	.401		
class 3, n (%)	10 (21.3)	12 (31.5)	0.58 (0.22 - 1.55)	.203		
Ki67, median (IQR)	80 (70 - 80)	80 (70 -90)		.652		
90-80%, n (%)	26 (55.3)	21 (55.3)	1.01 (0.42 - 2.36)	.584		
60-70%, n (%)	17 (36.2)	16 (42.1)	0.78 (0.32 - 1.87)	.368		
<60%, n (%)	4 (8.5)	1 (2.6)	3.44 (0.36 - 32.16)	.253		
History of other malignancies, n (%)	7 (14.9)	7 (18.4)	0.77 (0.24 - 2.44)	.441		
Diabetes mellitus, n (%)	9 (19.1)	10 (26.3)	0.66 (0.23 - 1.84)	.298		
Hypertension, n (%)	32 (68.1)	21 (55.3)	1.72 (0.71 - 4.18)	.161		
Anticoagulation, n (%)	4 (8.5)	6 (15.8)	0.49 (0.13 - 1.91)	.242		
Antiplatelet use, n (%)	7 (14.9)	9 (23.7)	0.56 (0.18 - 1.68)	.252		
Tumor volume (mL), median (IQR)	18.2 (8.1 -32.5)	15.2 (7.4 - 27.3)	–	.721	0.97 (0.95 - 1.02)	.269
Peritumoral edema (mL), median (IQR)	67.8 (25.4 - 122.3)	70.4 (26.5 - 92.5)	–	.998		
Total number of lesions, median (IQR)	1 (1-2)	1 (1 - 2)	–	.839		
Midline shift (mm), median (IQR),	2.5 (0 - 4.4)	2 (0 - 4.5)	–	.802		
Location						
supratentorial	43 (91.5)	34 (89.5)	1.26 (0.29 - 5.43)	.518		
infratentorial	0 (0)	1 (2.6)	–	.447		
both	4 (8.5)	3 (7.9)	1.08 (0.22 - 5.17)	.618		
Intracerebral hemorrhage on postoperative CT, n (%)	24 (51.1)	23 (60.5)	0.68 (0.28 - 1.61)	.257		
Revision surgery, n (%)	1 (2.1)	0 (0)	–	.552		
Non-space-occupying, n(%)	23 (48.9)	23 (60.5)	0.63 (0.26 - 1.48)	.198		
Abscess or Impaired wound healing, n (%)	1 (2.1)	2 (5.3)	0.39 (0.04 - 4.48)	.419		
Preoperative Dex, n (%)	8 (17)	9 (23.7)	0.66 (0.23 - 1.92)	.311		
Perioperative Dex before Systemic therapy, n (%)	45 (95.7)	35 (92.1)	1.92 (0.31 - 12.18)	. 399		
Perioperative Dex before systemic therapy (mg), median (IQR)	120 (62 - 168)	184 (157 - 233)	–	**<.0001**	1.2 (1.05 - 1.2)	**.002**
Duration of Dex administration (days) median (IQR)	9 (4 -12)	12.5 (8 - 16.5)	–	.**021**	0.98 (0.89 - 1.12)	.935
Systemic therapy					0.59 (0.23 - 1.55)	.286
R-MTX-base	14 (29.8)	13 (34.2)	0.82 (0.32 - 2.04)	.419		
Alternative regimens (intensive)	29 (61.7)	25 (65.8)	0.83 (0.34 - 2.04)	.436		
Other	4 (8.5)	0 (0)	–	.088		
The time between operation and chemoimmunotherapy (days), median (IQR)	12.5 (9 -15.8)	13.5 (9.3 - 17.8)	–	.363	0.98 (0.88 - 1.09)	.728
Complications, n (%)	21 (44.7)	13 (34.2)	1.55 (0.64 - 3.75)	.224	0.64 (0.19 - 2.04)	.447
Stem cell transplantation, n (%)	19 (40.4)	3 (7.9)	7.91 (2.12 - 29.49)	**.0005**	0.06 (0.01 - 0.37)	**.002**

Univariate and multivariate analyses were performed to identify predictors of overall survival. Continuous variables are presented as median (interquartile range, IQR), and categorical variables as number (percentage). Odds ratios (OR) are reported for univariate logistic regression, and hazard ratios (HR) for multivariate Cox proportional hazards regression. Statistically significant *p*-values (< 0.05) are highlighted in bold. Abbreviations: ASA - American Society of Anesthesiologists; BSA - body surface area; Dex - Dexamethasone; MSKCC - Memorial Sloan-Kettering Cancer Center, HR - hazard ratio; IQR - interquartile range; KPS - Karnofsky Performance Status; MTX - methotrexate; OR - odds ratio; OS - overall survival; PFS - progression-free survival; R-MTX - rituximab-methotrexate-based regimen; *p* - probability value.

### Cytolytic effect under dexamethasone exposure

Among 12 patients (11.4%) with available MRI data prior to chemotherapy, tumor cytoreduction following dexamethasone administration was observed in five (41.7%). The median cumulative dexamethasone dose prior to chemotherapy was 162 mg (IQR 114-240) in patients with radiographic cytoreduction compared to 58 mg (IQR 48-74) in those without measurable tumor shrinkage. Higher dexamethasone exposure was significantly associated with MRI-based tumor cytoreduction (*p* = .01).

## Discussion

In the present study, survival analysis demonstrated that administration of dexamethasone above the calculated threshold of 154 mg prior to chemoimmunotherapy was associated with significantly poorer outcomes. Multivariate analysis further demonstrated that higher cumulative dexamethasone exposure remained independently associated with poorer survival. Previous studies found no negative impact on survival from early corticosteroid administration in PCNSL (29). Consistent with this observation, our exploratory sensitivity analysis showed no significant differences in OS or PFS between patients with no pre-chemoimmunotherapy dexamethasone exposure and steroid-exposed patients within the low cumulative exposure range. However, given the very small number of unexposed patients, this comparison should be interpreted cautiously. These findings suggest that the association with outcome may depend more strongly on cumulative corticosteroid exposure than on corticosteroid administration per se. Accordingly, clinical management should move beyond the dichotomy of steroid use versus non-use, emphasizing instead quantitative optimization of dosing-maintaining the minimum effective amount required for symptomatic control without compromising long-term survival.

Survival outcomes in our cohort were within the range reported in prospective PCNSL studies, although direct comparisons should be interpreted cautiously because of differences in study design, patient selection, treatment intensity, and consolidation strategies. The 2-year OS was 47% in the PRIMAIN trial and approximately 69% with the MATRix regimen, compared with 76.2% in our cohort (30, 31). These differences should not be interpreted as evidence of superior efficacy in the present cohort, particularly given its retrospective design and the lower overall rate of autologous stem cell transplantation (ASCT) (24.7%).

It is noteworthy that ASCT was performed significantly more often in the favorable-prognosis group. Existing evidence indicates that ASCT is typically carried out after induction chemoimmunotherapy in patients who achieve a favorable response and are deemed fit for high-dose consolidation therapy (32). Consequently, patients with better chemosensitivity and clinical suitability undergo ASCT and subsequently demonstrate improved outcomes, emphasizing that ASCT is both a marker and a factor contributing to a favorable prognosis in PCNSL, and in many cases, this therapy is prescribed precisely because of a good response to initial treatment, a response potentially shaped by the patient’s dexamethasone exposure (20, 21).

Furthermore, beyond their anti-edema effects, corticosteroids exert a well-established cytolytic effect on PCNSL cells (33). Apoptosis induced by treatment with dexamethasone in lymphoid cells was one of the first models of apoptosis studied in detail due to DNA fragmentation into nucleosomal fragments (34). Сorticosteroid-induced apoptosis may result in marked depletion of neoplastic B cells and, in pretreated biopsy specimens, may produce predominantly reactive and resorptive tissue changes with only sparse residual tumor cells, thereby complicating histopathological diagnosis (16). These diagnostic alterations have been extensively characterized in large neuropathological series and may occasionally render the initial biopsy nondiagnostic (15). In our exploratory subgroup with serial pre-chemoimmunotherapy MRI (n = 12), five patients demonstrated radiographic tumor cytoreduction following dexamethasone exposure, with higher cumulative exposure associated with cytoreduction. Given the small sample size, this finding should be interpreted cautiously but is consistent with the known corticosteroid sensitivity of PCNSL cells.

These observations raise several non-mutually exclusive mechanisms that may contribute to the association between higher dexamethasone exposure and adverse outcome. Glucocorticoid resistance has been demonstrated in B-cell lymphoma, raising the possibility that early corticosteroid-induced cytoreduction could preferentially eliminate more steroid-sensitive cells while allowing less steroid-sensitive populations to persist, although direct evidence for such selection in PCNSL is lacking (35). In addition, dexamethasone has been shown to reduce blood–tumor barrier permeability in patients with brain tumors (36). Experimental data further suggest that corticosteroid exposure can reduce methotrexate delivery to tumor and brain tissue (37). Conversely, higher cumulative dexamethasone exposure may also represent a marker of greater baseline disease severity rather than a direct cause of inferior outcome. Although tumor volume, clinical status, and treatment interval did not independently account for the observed survival association in our multivariable analysis, residual confounding cannot be excluded.

Several clinical parameters have been consistently associated with improved remission rates and overall survival in PCNSL, including younger age, higher KPS or ECOG performance status ≤12, complete response to induction chemoimmunotherapy, and eligibility for high-dose methotrexate-based consolidation therapy or ASCT (38, 39). However, in our cohort, these classical prognostic indicators did not reach statistical significance, suggesting that patient condition alone does not fully account for survival variation. Similarly, the type of neurosurgical procedure was not associated with outcome in our cohort. However, given the small number of patients undergoing open biopsy or resection, procedure-specific effects cannot be reliably assessed. The Ki-67 proliferation index was also not significantly associated with outcome in our cohort, suggesting limited prognostic relevance of this parameter within the present dataset. Instead, higher cumulative dexamethasone exposure remained the only potentially modifiable variable independently associated with adverse outcome in the multivariable analysis. This finding implies that beyond conventional performance measures, the biological impact of pre-treatment dexamethasone may play a critical role in determining therapeutic outcomes in PCNSL.

Moreover, patients with higher cumulative dexamethasone exposure experienced a significantly longer interval between surgery and initiation of chemoimmunotherapy. Although this variable was not independently associated with survival in the multivariable analysis, prolonged diagnosis-to-treatment intervals have been linked to inferior outcomes in systemic diffuse large B-cell lymphoma (40, 41). In our cohort, a longer treatment interval may have contributed to greater cumulative corticosteroid exposure by prolonging the period during which dexamethasone was required for symptom control.

The identified 154-mg threshold should therefore be regarded as exploratory rather than as an established biological cutoff and requires prospective validation.

## Limitation

This study has several limitations. First, its retrospective design inherently carries the risk of selection and information bias and limits the ability to establish causal relationships between dexamethasone administration and survival outcomes. Second, the multicenter nature of the cohort implies a degree of heterogeneity in surgical techniques, perioperative management, and chemoimmunotherapy strategies, which could have influenced outcomes despite efforts to standardize data collection. Another limitation is the heterogeneity of systemic treatment across participating centers. Although treatment regimens were categorized according to composition and intensity and treatment category was included in the multivariable analysis, detailed information on treatment intensity, dose modifications, and completion of individual treatment cycles was not uniformly available. Third, Detailed immunohistochemical and molecular tumor profiles were not uniformly available across participating centers. Fourth, the subdivision of patients into remission and deceased groups has certain methodological constraints, given the long recruitment period. Some patients, particularly of advanced age, may have died of unrelated causes after achieving complete remission. Fifth, complete calculation of the International Extranodal Lymphoma Study Group score was not feasible for all patients because cerebrospinal fluid parameters and serum LDH values were not consistently available across participating centers, precluding comprehensive assessment of this established prognostic index (42). Finally, external validation in independent prospective cohorts is required to confirm the generalizability of our findings.

## Conclusion

Higher cumulative dexamethasone exposure before chemoimmunotherapy was associated with inferior survival outcomes in patients with PCNSL. The exploratory 154-mg threshold requires prospective and external validation, and the retrospective nature of this study precludes causal inference.

## Data Availability

The raw data supporting the conclusions of this article will be made available by the authors, without undue reservation.
