# Supplementary material for: Cumulative pre-chemoimmunotherapy dexamethasone dose is associated with adverse outcomes in primary central nervous system lymphoma
Source: Front Oncol. 2026 Sep 17;16:1905984. doi: 10.3389/fonc.2026.1905984 (PMC13627054; doi:10.3389/fonc.2026.1905984)
Supplement: Supplementary file 1 [file Table1.docx]

| Supplementary Table S1. Histopathological, immunohistochemical, and available molecular/reference-pathology findings in patients with retrievable detailed neuropathology reports. | | | | | |
| --- | --- | --- | --- | --- | --- |
| **Case ID** | **Histomorphology** | **B-cell phenotype** | **Additional IHC** | **Ki-67** | **Final diagnosis / relevant comments** |
| P01 | Dense raster-like infiltrating neoplasia with high cellularity, moderately pleomorphic nuclei, mitoses, and focal necrosis. | LCA+,  CD20+ | CD3/CD4/CD8 reactive background; MUM1+; CD138− | ~80% | Intracerebral aggressive B-cell NHL corresponding to DLBCL. |
| P02 | Raster-like high-cellularity tumor with moderately pleomorphic nuclei; apoptosis; focal necrosis. | CD20+, CD79a+ | MUM1+; CD3/CD4/CD8 reactive background; CD138 rare/negative | ~70% | Intracerebral aggressive B-cell NHL corresponding to DLBCL. |
| P03 | Diffuse large-cell lymphoid infiltrate in cerebral tissue. | CD20+ | CD3 reactive; CD10−; Cyclin D1− | ~80% | Diffuse large B-cell lymphoma compatible with primary CNS lymphoma. |
| P04 | High-grade B-cell lymphoma infiltrating cerebral tissue. | CD20+ | CD3−; CD10−; CD30−; Cyclin D1− | ~70% | High-grade B-cell lymphoma compatible with primary CNS DLBCL. |
| P05 | Cell-rich atypical lymphoid population with prominent perivascular/angiocentric arrangement. | CD45+,  CD20+ | CD3/CD5 reactive T cells; CD10−; CD21−; BCL6+; MUM1+ | >80% | DLBCL; morphology and immunophenotype compatible with primary CNS lymphoma, non-GCB phenotype described. |
| P06 | Cell-dense partly altered tumor tissue with pleomorphic cells, mitoses/apoptosis, focal necrosis. | CD20+ | CD3/CD4/CD8 reactive background; CD138− | ~80% | DLBCL in brain, compatible with primary CNS lymphoma if systemic disease is excluded |
| P07 | Large atypical lymphoid infiltrates in brain tissue. | CD45+,  CD20+ | CD3−; additional exclusion markers reported | ~80% | Diffuse large B-cell lymphoma; primary CNS lymphoma considered after staging. |
| P08 | Highly pleomorphic raster-like tumor with dyscohesive cells, mitoses and apoptosis. | CD20+, CD79a+ | MUM1 focal (~40%); CD3+ T-cell background; | ~70% | Diffuse large B-cell lymphoma; primary CNS lymphoma considered after staging. |
| P09 | Extensive infiltrates of neoplastic lymphoid cells, focally perivascular; large pleomorphic nuclei, mitoses and apoptosis. | CD20+ | CD10−; BCL6 <30%+; IRF4/MUM1 diffuse+; EBV-LMP1−; CD3+ reactive T cells | >70% | DLBCL in brain, compatible with primary CNS lymphoma if systemic disease is excluded |
| P10 | Perivascular infiltrates of atypical lymphoid cells. | CD45+, CD20+ on diagnostic repeat biopsy | CD10−; CD3 reactive | ~70% | Diffuse large B-cell lymphoma compatible with primary CNS lymphoma. |
| P11 | Initial biopsy: reactive CNS tissue with sparse predominantly CD8+ lymphocytes; repeat biopsy: dense high-grade B-cell tumor. | CD20+ | CD3/CD4/CD8 reactive background; CD68 macrophages | ~80% | B-cell lymphoma established on repeat biopsy after nondiagnostic/reactive first biopsy. |
| P12 | High-grade B-cell lymphoma morphology. | CD20+ | TdT−; CD34−; Cyclin D1−; CD5− on supplemental IHC | ~80% | High-grade B-cell lymphoma of DLBCL type; lymphoblastic B-cell lymphoma excluded. |
| P13 | High-grade large B-cell lymphoma infiltrating brain tissue. | CD20+ | BCL2+; CD10−; Cyclin D1− | ~90% | Cerebral high-grade B-cell lymphoma; |
| P14 | High-grade B-cell lymphoma morphology. | CD20+, CD79a+ | TdT−; CD34−; Cyclin D1−; | ~80% | High-grade B-cell lymphoma of DLBCL type; lymphoblastic B-cell lymphoma excluded. |
| P15 | Predominantly dense diffuse brain infiltrates of high-grade blastoid B-cell lymphoma. | CD20+, CD79a+ | CD10−; BCL2 partly+; BCL6+; MUM1 strong+; CD3 reactive T cells | >80% | DLBCL; immunophenotype supports primary CNS lymphoma after staging. |
| P16 | Diffuse infiltrating high-grade B-cell NHL in brain parenchyma. | CD20+, CD79a+ | CD10−; BCL6+; IRF4/MUM1+; BCL2 strong+; c-MYC >50% | ~80% | Diffuse large B-cell lymphoma; phenotype compatible with primary CNS lymphoma after staging. |
| P17 | Diffuse infiltrating high-grade B-cell NHL in brain parenchyma. | CD20+, CD79a+ | — | ~80% | High-grade B-cell lymphoma / DLBCL; primary CNS lymphoma considered possible. |
| P18 | Diffuse malignant B-cell lymphoma with cerebral involvement. | CD20+ | BCL2+; CD10−; Cyclin D1−; CD3/CD5− in tumor population | ~50% | High-grade B-cell lymphoma / DLBCL; primary CNS lymphoma considered possible. |
| P19 | Dense cohesive/angiocentric atypical lymphoid proliferation with mitoses and apoptosis. | CD20+ | CD10−; BCL6+; IRF4/MUM1+; BCL2 strong+; c-MYC >50% | ~80% | Diffuse large B-cell lymphoma; phenotype compatible with primary CNS lymphoma after staging. |
| P20 | Diffuse malignant B-cell lymphoma with cerebral involvement. | CD20+, CD79a+, PAX5+ | BCL2+; CD10−; CD30−; partial Cyclin D1 nuclear positivity | ~60% | Malignant B-cell lymphoma compatible with primary CNS DLBCL; |
| P21 | Neoplastic lymphoid infiltrate with extensive necrosis; residual tumor centered on vessels; abundant apoptotic bodies after steroid pretreatment. | CD20+, CD79a+ | CD10−; Cyclin D1−; CD30−; BCL6 partial+ | ~90% | High-grade B-cell NHL / DLBCL; steroid-reduced infiltrate compatible with primary CNS lymphoma. |
| P22 | Cell-dense lymphoblastic proliferation with focal necrosis and abundant apoptosis. | CD20+, CD79a+ | BCL6 partial+; BCL2 strong+; MUM1 >50%+; CD10−; AE1/3−; GFAP background | >50% | High-grade B-cell NHL, primarily DLBCL; primary CNS lymphoma possible after staging. |
| P23 | Dense diffuse high-grade B-cell lymphoma infiltrates in brain. | CD20+, CD79a+ | CD10−; BCL6+; MUM1 strong+; BCL2 partial+; CD3 reactive T cells | ~70% | DLBCL compatible with primary CNS lymphoma. |
| P24 | Large pleomorphic lymphoid cells with mitoses, necrosis and apoptosis. | CD20+, CD79a+ | BCL2+; CD5−; CD10− | ~70–80% | Diffuse large B-cell lymphoma compatible with primary CNS lymphoma. |
| P25 | Extensive perivascular and sheet-like blastoid lymphoid infiltrates; mitoses and abundant apoptosis; histiocytes in areas possibly modified by dexamethasone. | CD20+, CD79a+ | CD10−; BCL6+; IRF4/MUM1+; CD3+ reactive T cells | ~70% | High-grade B-cell lymphoma / DLBCL compatible with primary CNS lymphoma; steroid-associated changes explicitly described. |
| P26 | High-grade diffuse B-cell lymphoma with diffuse growth pattern. | CD20+, CD79a+ | — | ~80% | High-grade B-cell lymphoma / DLBCL; |
| P27 | Brain tissue with infiltrates of medium-large atypical lymphoid cells. | LCA+, CD20+, CD79a+ | CD10−; Cyclin D1− | >80% | Diffuse large B-cell lymphoma in brain; primary CNS lymphoma considered possible after staging. |
| P28 | Large atypical lymphoid cells, partly concentrically/perivascular around vessels. | CD20+, CD79a+ | CD10+; additional CD5/Cyclin D1 results negative in addendum; c-MYC >50%+ | >80% | DLBCL with CD10 expression; systemic lymphoma required clinical exclusion. |
| P29 | Predominantly resorptive-inflammatory/steroid-altered brain tissue with residual perivascular lymphoid infiltrates. | CD20+, CD79a+ | CD10−; Cyclin D1− | ~50% | Steroid-modified DLBCL / primary CNS lymphoma considered. |
| P30 | Cell-rich blastoid tumor with medium-sized round to pleomorphic cells, numerous mitoses and focal vessel association. | CD20+, CD79a+ | BCL6+; CD10−; CD5−; Cyclin D1−; CD3+ bystander T cells | ~50% | High-grade B-cell NHL of DLBCL type, compatible with primary CNS lymphoma. |
| P31 | High-grade diffuse B-cell lymphoma with diffuse growth pattern. | CD20+, CD79a+ | MUM1 strong+; BCL2 strong+; BCL6 >80%+; CD10−; Cyclin D1−; CD3 reactive | >70% | Diffuse large B-cell lymphoma; double-/triple-hit-type rearrangements excluded. |
| P32 | Diffuse/raster-like and perivascular blastoid lymphoid infiltrates with numerous mitoses and apoptotic bodies. | CD20+, CD79a+ | CD10−/positive in addendum depending sampled material; BCL6+; IRF4/MUM1+; Cyclin D1−; CD30− | ~60–70% | Diffuse large B-cell lymphoma in brain; primary CNS lymphoma considered possible after staging. |
| P33 | Extensive raster-like infiltrates of atypical large lymphoid cells with prominent nuclei, mitoses and focal necrosis. | CD20+, CD79a+ | CD10−; BCL6+; IRF4/MUM1+; Cyclin D1−; CD3 reactive | ~70% | High-grade B-cell lymphoma / DLBCL; findings compatible with primary CNS lymphoma after staging. |
| P34 | Dense partly diffuse/raster-like infiltrates of atypical lymphoid cells. | LCA+,  CD20+, CD79a+ | IRF4/MUM1+; BCL6 partly+; CD10−; BCL2 strong+; c-MYC >50%; CD3/CD5 reactive T cells | ~70% | High-grade B-cell NHL / DLBCL; primary CNS lymphoma considered possible after staging. |
| P35 | Diffuse perivascularly accentuated infiltrates of atypical lymphoid cells with mitoses, apoptosis and focal necrosis. | LCA+, CD20+ | BCL6 focal+; CD10−; cytokeratin AE1/3−; GFAP limited to glial background | ~60% | Diffuse large B-cell lymphoma; immunophenotype considered highly compatible with primary CNS lymphoma after staging. |
| P36 | Extensive perivascular large-cell lymphoid tumor with necrosis in multiple biopsy portions. | CD20+ | BCL6+; MUM1+; p53 moderate+; CD10−; AE1/3−; GFAP−; IDH1−; CD3/CD5 reactive T cells | ~80% | Diffuse large B-cell lymphoma in diagnostic biopsy portions; primary CNS lymphoma compatible after staging. |
| P37 | Dense atypical large-cell lymphoid infiltrate adjacent to a venous vessel; numerous apoptotic bodies. | CD20+, CD79a+ | CD10+; BCL6 ~60%+; MUM1 sparse+; Cyclin D1−; CD3/CD5 reactive T cells | ~60% | Diffuse large B-cell lymphoma, germinal-center phenotype; extracerebral lymphoma to be excluded clinically. |
| P38 | Primary cerebral B-cell lymphoma morphology; partial lymphoplasmacytic/plasmacytic differentiation described. | CD20+ | - | ~90% | Primary cerebral B-cell lymphoma considered; staging required for final classification. |
| P39 | Relatively dense lymphoid infiltrates containing larger blastoid cells with hyperchromatic nuclei and prominent nucleoli. | CD20+, CD79a+, MUM1+ | MUM1 strong+; BCL6 weak/partial+; CD10−; Cyclin D1−; CD23−; CD3/CD5 small T cells | ~60% | Diffuse large B-cell lymphoma, non-germinal-center phenotype. |
| P40 | Extensive raster-like infiltrates of atypical lymphoid cells with vesicular nuclei, prominent nucleoli, mitoses and focal necrosis. | CD20+, CD79a+ | BCL6 partly+; CD10−; Cyclin D1−; CD3/CD5 reactive T cells | ~90% | Diffuse large B-cell lymphoma, non-germinal-center phenotype. |
| P41 | Very dense diffuse lymphoid infiltrate with partial perivascular/vessel-wall infiltration, necrosis, mitoses and apoptotic bodies. | CD20+, CD79a+ | CD10+; BCL6+; CD5−; CD30−; Cyclin D1−; CD3 reactive T cells | ~70% | Diffuse large B-cell lymphoma in brain; |
| P42 | Large B-cell lymphoma infiltrating brain tissue at two sites, with necrosis. | CD20+, CD79a+ | IDH1−; CD3 scattered T cells | ~80% | Diffuse large B-cell lymphoma in brain; |
| P43 | Partly perivascular and partly diffuse infiltrates of atypical large lymphoid cells with necrosis, mitoses and apoptotic bodies. | CD20+, CD79a+ | BCL6+; CD10−; CD5−; CD30−; very rare Cyclin D1+ cells; CD3 reactive T cells | ~70% | Diffuse large B-cell lymphoma with reactive T-cell infiltrate; |
| P44 | One specimen showed reactive brain tissue; tumor specimen showed disturbed architecture with perivascular and diffuse large B-cell infiltration. | CD20+, CD79a+ | BCL6+; CD5−; CD10−; Cyclin D1−; IDH1−; CD3 reactive T cells | ~80% | Diffuse large B-cell lymphoma with reactive T-cell infiltrate; |
| P45 | Initial biopsy: reactive CNS tissue with sparse predominantly CD8+ lymphocytes; repeat biopsy: dense high-grade B-cell tumor. | CD20+, CD79a+ | CD3/CD4/CD8 reactive background; CD68 macrophages | ~80% | B-cell lymphoma established on repeat biopsy after nondiagnostic/reactive first biopsy. |
| P46 | Large B-cell lymphoma infiltrating brain tissue at three sites, with necrosis. | LCA+,  CD20+, CD79a+ | — | ~80% | High-grade B-cell lymphoma / DLBCL; findings compatible with primary CNS lymphoma after staging. |
| P47 | Diffuse large-cell lymphoid infiltrate in cerebral tissue. | CD20+ | CD3 reactive; CD10−; | ~85% | Diffuse large B-cell lymphoma compatible with primary CNS lymphoma. |
| P48 | Extensive raster-like infiltrates of atypical large lymphoid cells with prominent nuclei, mitoses and focal necrosis. | CD20+, CD79a+ | CD10−; BCL6+; IRF4/MUM1+; Cyclin D1−; CD3 reactive | ~70% | High-grade B-cell lymphoma / DLBCL; findings compatible with primary CNS lymphoma after staging. |
| P49 | Diffuse malignant B-cell lymphoma with cerebral involvement. | CD20+, CD79a+, | BCL2+; CD10−; CD30−; partial Cyclin D1 nuclear positivity | ~60% | Malignant B-cell lymphoma compatible with primary CNS DLBCL; |
| P50 | Raster-like high-cellularity tumor with moderately pleomorphic nuclei and apoptosis | CD20+, CD79a+ | MUM1+; CD3/CD4/CD8 reactive background; CD138- | ~70% | Intracerebral aggressive B-cell NHL corresponding to DLBCL. |
| P51 | Large atypical lymphoid infiltrates in brain tissue. | CD45+,  CD20+ | CD3−; additional exclusion markers reported | ~80% | Malignant B-cell lymphoma compatible with primary CNS DLBCL; |
| P52 | High-grade diffuse B-cell lymphoma with diffuse growth pattern. | CD20+, CD79a+ | CD10−; MUM1 +; BCL2 s+; BCL6 >80%+; Cyclin D1−; | >70% | High-grade B-cell lymphoma / DLBCL; findings compatible with primary CNS lymphoma after staging. |
| P53 | Primary cerebral B-cell lymphoma morphology; | CD20+  CD79a+ | CD10−; Cyclin D1− | ~85% | Primary cerebral B-cell lymphoma considered; |
