# Supplementary material for: Cumulative pre-chemoimmunotherapy dexamethasone dose is associated with adverse outcomes in primary central nervous system lymphoma
Source: Front Oncol. 2026 Sep 17;16:1905984. doi: 10.3389/fonc.2026.1905984 (PMC13627054; doi:10.3389/fonc.2026.1905984)
Supplement: Supplementary file 2 [file Table2.docx]

| Supplementary Table S2. Characteristics and outcomes of patients with no pre-chemoimmunotherapy dexamethasone exposure compared with steroid-exposed patients within the low cumulative exposure range. | | | |  |
| --- | --- | --- | --- | --- |
|  | **No dexamethasone (0 mg)** | **Low cumulative exposure (1–154 mg)** | **p-value** |  |
| **Count** | **5** | **49** |  |  |
| Sex, male, n (%) | 1 (20.0) | 28 (57.1) | .170 |  |
| Age, median (IQR), years | 68 (54–78) | 69 (63–73) | .929 |  |
| BSA, median (IQR), m² | 1.9 (1.8–2.0) | 2.0 (1.8–2.1) | .630 |  |
| **Operation type** |  |  |  |  |
| Stereotactic biopsy, n (%) | 5 (100.0) | 43 (87.8) | 1.000 |  |
| Open biopsy, n (%) | 0 (0.0) | 1 (2.0) | 1.000 |  |
| Resection, n (%) | 0 (0.0) | 5 (10.2) | 1.000 |  |
| Re-biopsy, n (%) | 0 (0.0) | 3 (6.1) | 1.000 |  |
| Preoperative ASA, median (IQR) | 3 (2–3) | 2 (2–3) | .347 |  |
| ASA 1–2, n (%) | 2 (40.0) | 30 (61.2) | .388 |  |
| ASA 3–4, n (%) | 3 (60.0) | 19 (38.8) | .388 |  |
| Preoperative KPS, median (IQR) | 70 (60–80) | 70 (60–90) | .530 |  |
| KPS 80–100, n (%) | 2 (40.0) | 20 (40.8) | 1.000 |  |
| KPS 60–70, n (%) | 2 (40.0) | 26 (53.1) | .663 |  |
| KPS <60, n (%) | 1 (20.0) | 3 (6.1) | .330 |  |
| **MSKCC prognostic score** |  |  |  |  |
| Median (IQR) | 2 (2–3) | 2 (2–3) | .435 |  |
| Class 1, n (%) | 0 (0.0) | 4 (8.2) | 1.000 |  |
| Class 2, n (%) | 3 (60.0) | 32 (65.3) | 1.000 |  |
| Class 3, n (%) | 2 (40.0) | 13 (26.5) | .610 |  |
| **Ki-67** |  |  |  |  |
| Median (IQR), % | 70 (70–80) | 80 (70–80) | .742 |  |
| ≥80%, n (%) | 2 (40.0) | 26 (53.1) | .663 |  |
| 60–70%, n (%) | 3 (60.0) | 20 (40.8) | .640 |  |
| <60%, n (%) | 0 (0.0) | 3 (6.1) | 1.000 |  |
| History of other malignancy, n (%) | 1 (20.0) | 9 (18.4) | 1.000 |  |
| Diabetes mellitus, n (%) | 2 (40.0) | 9 (18.4) | .266 |  |
| Hypertension, n (%) | 3 (60.0) | 33 (67.3) | 1.000 |  |
| Anticoagulation, n (%) | 0 (0.0) | 7 (14.3) | 1.000 |  |
| Antiplatelet use, n (%) | 1 (20.0) | 6 (12.2) | .515 |  |
| Tumor volume (mL), median (IQR) | 3.9 (3.3–6.9) | 14.6 (5.2–19.6) | **.032** |  |
| Peritumoral edema (mL), median (IQR) | 20.6 (16.8–58.6) | 78.9 (32.8–122.5) | .149 |  |
| Total number of lesions, median (IQR) | 2 (1–2) | 1 (1–3) | .884 |  |
| Midline shift (mm), median (IQR) | 1.8 (1.5–3.0) | 1.0 (0–4.0) | .446 |  |
| **Location** |  |  |  |  |
| Supratentorial, n (%) | 5 (100.0) | 43 (87.8) | 1.000 |  |
| Infratentorial, n (%) | 0 (0.0) | 0 (0.0) | — |  |
| Both, n (%) | 0 (0.0) | 6 (12.2) | 1.000 |  |
| Intracerebral hemorrhage on postoperative CT, n (%) | 4 (80.0) | 24 (49.0) | .353 |  |
| Revision surgery, n (%) | 0 (0.0) | 1 (2.0) | 1.000 |  |
| Non-space-occupying hemorrhage, n (%) | 4 (80.0) | 23 (46.9) | .351 |  |
| Preoperative dexamethasone, n (%) | 0 (0.0) | 11 (22.4) | .571 |  |
| Postoperative dexamethasone before systemic therapy, n (%) | 0 (0.0) | 49 (100.0) | **<.001** |  |
| Cumulative dexamethasone before systemic therapy, median (IQR), mg | 0 (0–0) | 90 (64–124) | **<.001** |  |
| Duration of dexamethasone administration, median (IQR), days | 0 (0–0) | 8 (4–12) | **<.001** |  |
| **Systemic therapy** |  |  |  |  |
| R-MTX-base, n (%) | 2 (40.0) | 15 (30.6) | .645 |  |
| Alternative regimens (intensive), n (%) | 3 (60.0) | 31 (63.3) | 1.000 |  |
| Other, n (%) | 0 (0.0) | 3 (6.1) | 1.000 |  |
| Time between operation and chemoimmunotherapy, median (IQR), days | 12 (12–13) | 11 (8–16) | .939 |  |
| Stem cell transplantation, n (%) | 1 (20.0) | 16 (32.7) | 1.000 |  |
| 1-year OS, n (%) | 4 (80.0) | 47 (95.9) | .257 |  |
| 2-year OS, n (%) | 4 (80.0) | 47 (95.9) | .257 |  |
| Deceased cases, n (%) | 1 (20.0) | 7 (14.3) | .567 |  |
|  |  |  |  |  |
| *Continuous variables were compared using the Mann–Whitney U test and categorical variables using Fisher’s exact test. The 0-mg group comprised patients with no dexamethasone exposure before initiation of chemoimmunotherapy; the comparison group included only steroid-exposed patients with cumulative exposure of 1–154 mg. Given the small number of unexposed patients (n=5), these analyses are exploratory.* | | | |  |
|  |  |  |  |  |
|  |  |  |  |  |
